# Trends and disparities in mortality associated with peripheral artery disease and hyperlipidemia, 1999–2024

**DOI:** 10.1038/s41598-025-29224-7

**Published:** 2025-11-22

**Authors:** Saifullah Khan, Muhammad Hassan, Muhammad Hussain, Hassan Abdul Aziz Dhedhi, Wania Fatima, Javeria Nawaz, Aiza Ahsan, F. N. U. Pirih, Nisha Khalid, Mariam Qaisar, Ahmad Anees Qureshi, Maria Baig, Sherif Eltawansy, Muhammad Khalid Afridi, Preet Memon, Saad Ahmed Waqas, Gregg C. Fonarow, Raheel Ahmed, Hasibullah Aminpoor

**Affiliations:** 1 Department of Medicine, Dow University of Health Sciences, Karachi, Pakistan; 2 Department of Medicine, People’s University of Medical and Health Sciences, Shaheed Benazirabad, Pakistan; 3 Department of Medicine, Jersey Shore University Medical Center, Neptune, NJ USA; 4 Ahmanson-UCLA Cardiomyopathy Center, Ronald Reagan-UCLA Medical Center, Los Angeles, CA USA; 5 Department of Cardiology, Newcastle Upon Tyne Hospitals, NHS Foundation Trust, Newcastle Upon Tyne, UK; 6 Faculty of Medicine, Kabul University of Medical Sciences “Abu Ali Ibn Sina”, Ata Turk Avenue, Jamal Mena, 3rd District, 1001 Kabul, Afghanistan

**Keywords:** Cardiology, Diseases, Health care, Medical research, Risk factors

## Abstract

Peripheral artery disease (PAD) with hyperlipidemia adds significantly to the national burden of cardiovascular disease. However, population-level trends in mortality for these conditions are not well understood. We used nationally representative data to investigate long-term temporal patterns and disparities in PAD and hyperlipidemia related mortality in the United States. This descriptive analysis analyzed death records from the CDC WONDER database from 1999 to 2024. The age-adjusted mortality rates (AAMRs) per 100,000 people were calculated for each demographic and geographic strata. AAMRs were standardized to 2000 U.S. standard population. Temporal trends in AAMRs were analyzed using Joinpoint regression to estimate the average annual percent change (AAPC) with 95% confidence intervals (CIs) and *p* value. Between 1999 and 2024, a total of 148,416 PAD with hyperlipidemia–related deaths were recorded among U.S. adults ≥ 25 years. The national AAMR rose from 0.74 in 1999 to 3.92 in 2024, corresponding to an overall AAPC of 6.32. Both sexes experienced increasing mortality, though men had higher rates and faster growth compared with women. By race/ethnicity, AAMRs increased across all groups, with the steepest relative rise among Hispanic individuals, followed by Non-Hispanic (NH) Black, NH White, and NH Asian or Pacific Islander populations. Geographic disparities were observed, with state-level AAMRs ranging from 0.92 in Georgia to 5.06 in Vermont, and the highest regional mean AAMR in the West (2.93). Finally, rural areas consistently exhibited higher mean AAMRs than urban areas, with slightly steeper increase over time. AAMRs related to PAD and hyperlipidemia have increased more than fivefold, with disproportionately higher rates among men, Hispanic populations, residents of Western regions, and those in rural areas. These patterns suggest widening disparities in vascular health and underscore the need for strengthened preventive efforts, improved lipid management, and more equitable access to cardiovascular care.

**Supplementary Information:**

The online version contains supplementary material available at 10.1038/s41598-025-29224-7.

## Introduction

Peripheral artery disease (PAD) can be defined as a condition that develops when atherosclerosis, or plaque accumulation, occurs in peripheral arteries, which carry blood away from the heart and toward the peripheral regions of the body, narrowing them and limiting blood flow to the limbs and other parts of the body^1^. PAD affects approximately 200 million people globally and is frequently asymptomatic or misattributed to musculoskeletal causes, delaying detection and treatment^2^. In contrast, hyperlipidemia refers to a variety of conditions associated with elevated lipid levels in the bloodstream^3^. Approximately 25 million adults in the United States have total cholesterol levels over 240 mg/dL, which places them in the hyperlipidemic range^4^. Lower extremity PAD is the third most common cause of atherosclerotic cardiovascular events, following coronary artery disease and stroke^4^. Despite statin availability, guideline-concordant lipid management in PAD remains suboptimal in practice, with undertreatment linked to higher limb events and mortality^5^.

High cholesterol is a major contributor to peripheral artery disease^6^. LDL-C and other lipid abnormalities—such as high triglycerides, low HDL-C, elevated apolipoprotein B, and lipoprotein(a)—are linked to PAD and adverse limb outcomes, independent of traditional risks^7–9^. Dyslipidemia, diabetes, obesity, genetic hypercholesterolemia, and physical inactivity further contribute to PAD^9^. Conversely, healthy lifestyle practices and appropriate medical management can help prevent its onset^10,11^.

This study utilizes CDC Wonder database, which provides nationwide, standardized publicly available mortality data, making it ideal for evaluation of population-level mortality trends associated with PAD and hyperlipidemia. Additionally, this study emphasizes mortality trends derived from death certificates to capture population-level burden rather than clinical incidence. While individual effects of PAD and hyperlipidemia on patient outcomes are well-documented, there is still a considerable lack of literature examining their combined impact on mortality trends. Leveraging data from the CDC WONDER database (1999–2024), this analysis examines mortality trends among individuals with PAD and hyperlipidemia. We believe that this grouping represents a particularly high-risk phenotype, influenced by shared atherogenic processes and inconsistent adherence to lipid-lowering treatments. These findings may help with early detection, guideline-directed treatment, and policy actions aimed at lowering excess cardiovascular and limb mortality.

## Methods

### Study design and data source

This present study leveraged database of Centers for Disease Control and Prevention’s Wide-ranging Online Data for Epidemiologic Research (CDC WONDER)^12^ database, covering the time period from 1999 to 2024. The data were derived from U.S. death certificates as part of the Multiple Cause-of-Death Public Use data set, which include information from all 50 states and the District of Columbia. The database has been extensively used in prior epidemiologic studies for analyzing nationwide mortality trends.

### Study population

Mortality data were obtained using International Classification of Diseases, 10th Revision (ICD-10) codes. Death certificates listing PAD as either the underlying or as a contributing cause of death were identified with ICD-10 codes (E10.5, E11.5, E12.5, E13.5, E14.5, I70.0–I70.2, I70.8–I70.9, I71.1–I71.6, I71.8–I71.9, I72.1–I72.4, I72.8–I72.9, I73.8–I73.9, I74.0, I74.1–I74.5, I74.8–I74.9, and I77.8–I77.9) in patients ≥ 25 years of age^13^. The code E78 refers to hyperlipidemia^14^. Mortality events were considered if the condition of interest appeared anywhere on the death certificate, whether as a primary or contributing cause of death.

### Data abstraction

Variables collected included year of death, demographic characteristics (sex, race/ethnicity), location of death, geographic region, state, and urban–rural classification. Place of death was categorized into medical facilities (including outpatient, emergency room, inpatient, death on arrival, or status unknown), home, hospice, and long-term care facilities. Race/ethnicity were categorized as NH White, NH Black, Hispanic, and NH Asian or Pacific Islander. Urban–rural grouping was determined using the National Center for Health Statistics Urban–Rural Classification Scheme based on 2013 U.S. Census data^15^. Geographic regions were designated in accordance with the U.S. Census Bureau’s regional designations (Northeast, Midwest, South, and West).

### Statistical analysis

We calculated Age-adjusted mortality rates (AAMRs) per 100,000 population by year, sex, race/ethnicity, state, and urban–rural status, along with 95% confidence intervals (CIs). National temporal patterns in AAMRs were assessed using the Joinpoint Regression Program version 5.4.0, National Cancer Institute^16^, which identifies statistically significant changes in trends over time using log-linear models. Annual percent change (APC) and corresponding 95% CIs were reported. The APC measures the yearly rate of change in mortality within a given period, while the AAPC summarizes these changes to show overall long-term trend by providing a weighted average across multiple time segments. These indicators are integral to population-level analyses using CDC WONDER mortality data, as they account for non-linear trends over time and quantify the change in patterns over time. Statistical significance defined as a two-tailed *P*-value < 0.05.

## Results

Between 1999 and 2024, PAD with hyperlipidemia accounted for a total of 148,416 deaths among adults aged 25 years and more in the United States (Supplemental Table 1). These deaths were prevalent across various different places, with the leading most occurring in decedent’s homes [38.2%], followed by medical facilities [34.6%], [19.7%] in the nursing home/ long term care facilities, [3.1%] in hospice facilities, and [4.2%] at other locations (Supplemental Table 2).

**Table 1:** Annual percentage change for peripheral artery disease-related deaths among U.S. adults aged ≥ 25 years with hyperlipidemia, 1999 to 2024.

Demographics	Trend 1		Trend 2		Trend 3		Trend 4		Trend 5		Trend 6	
	Year	AAPC	Year	APC	Year	APC	Year	APC	Year	AAPC	Year	APC
Overall	1999–2024	6.32* (5.65 to 7.00)	1999–2001	35.40* (20.27 to 47.96)	2001–2006	6.95* (3.18 to 10.80)	2006–2018	1.73* (0.02 to 2.20)	2018–2021	12.92* (8.80 to 15.30)	2021–2024	0.68 (−4.03 to 3.18)
*Sex*												
Female	1999–2024	4.89* (4.39 to 5.69)	1999–2005	10.42* (6.59 to 16.82)	2005–2018	1.34 (− 0.39 to 2.23)	2018–2021	13.69* (7.66 to 17.03)	2021–2024	1.38 (−4.27 to 5.06)		
Male	1999–2024	6.40* (5.72 to 7.29)	1999–2001	35.37* (19.77 to 51.91)	2001–2005	8.84* (3.05 to 12.76)	2005–2018	2.15 (− 0.53 to 2.67)	2018–2021	11.92* (7.73 to 14.31)	2021–2024	−0.23 (−4.16 to 2.13)
*Race*												
Non-Hispanic Asian or Pacific Islander	1999–2024	3.93* (3.38 to 4.77)										
Non-Hispanic Black	1999–2024	6.34* (5.68 to 6.90)	1999–2004	19.16* (13.77 to 28.43)	2004–2018	2.50* (1.54 to 3.18)	2018–2021	15.66* (10.41 to 18.50)	2021–2024	−0.33 (−5.32 to 2.70)		
Non-Hispanic White	1999–2024	6.82* (6.20 to 7.67)	1999–2001	35.45* (21.43 to 45.79)	2001–2006	7.15* (3.49 to 11.03)	2006–2018	1.53 (− 0.43 to 2.07)	2018–2021	12.79* (8.69 to 15.19)	2021–2024	1.42 (−2.93 to 3.73)
Hispanic	1999–2024	7.12* (5.92 to 8.52)	1999–2001	40.45* (11.88 to 65.82)	2001–2017	3.95* (2.41 to 4.72)	2017–2021	12.74* (9.08 to 17.39)	2021–2024	−1.96 (−6.82 to 1.65)		
*Census Region*												
Northeast	1999–2024	6.50* (5.78 to 7.62)	1999–2001	27.36* (11.24 to 46.14)	2001–2012	5.43* (4.06 to 6.91)	2012–2017	− 0.22 (− 6.41 to 2.28)	2018–2020	15.80* (9.81 to 20.27)	2020–2024	2.06 (−2.48 to 4.32)
Midwest	1999–2024	5.45* (4.61 to 6.45)	1999–2001	39.15* (20.33 to 58.65)	2001–2006	8.05* (1.89 to 12.54)	2006–2018	− 0.07 (− 4.25 to 0.75)	2018–2021	11.62 (5.03 to 15.28)	2021–2024	−1.39 (−8.32 to 2.93)
South	1999–2024	5.67* (5.23 to 6.32)	1999–2005	12.10 * (8.95 to 16.75)	2005–2017	0.60 (− 0.49 to 1.40)	2017–2021	14.72* (11.53 to 19.86)	2021–2024	2.42 (−1.74 to 5.58)		
West	1999–2024	6.62* (5.35 to 8.65)	1999–2001	45.05* (12.13 to 83.35)	2001–2024	3.81* (3.25 to 4.32)						
*Urbanization*												
Rural	1999–2024	7.07* (6.34 to 8.11)	1999–2002	25.90* (17.36 to 43.94)	2002–2008	5.73* (2.91 to 9.38)	2008–2018	0.73 (− 2.67 to 1.52)	2018–2020	18.35* (9.93 to 23.80)		
Urban	1999–2024	6.90* (6.06 to 7.64)	1999–2001	3.04* (19.45 to 45.61)	2001–2006	6.68* (2.94 to 10.81)	2006–2018	1.90 (− 1.26 to 2.46)	2018–2020	14.27* (8.35 to 19.63)		

* indicate statistically significant trend.

### Annual trends for peripheral artery disease with hyperlipidemia-related AAMR

**Fig. 1:**
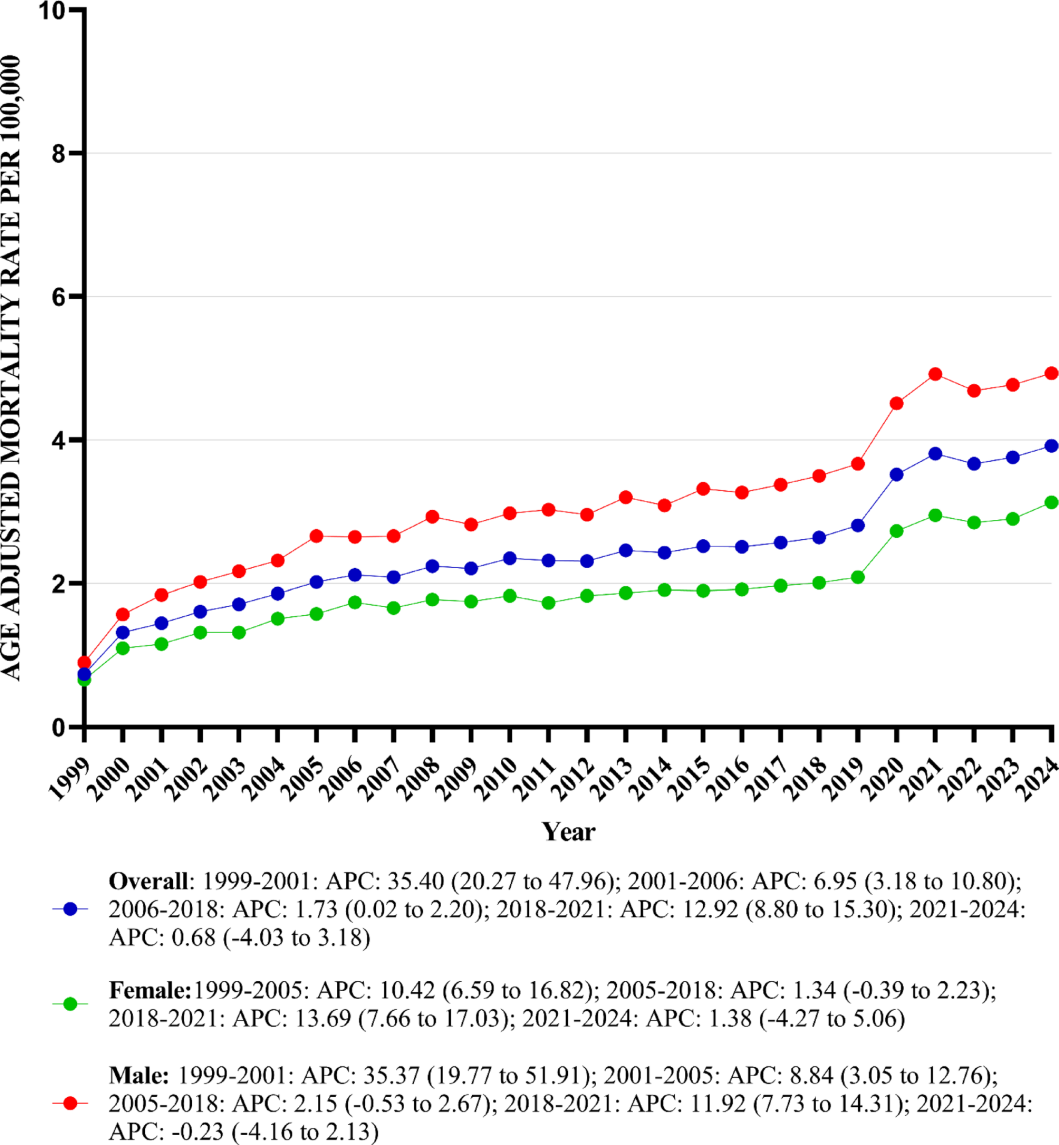
Peripheral artery disease with hyperlipidemia AAMR stratified by sex and overall, per 100,000 population.

The overall AAMR for peripheral artery disease with hyperlipidemia-related deaths among adults increased from 0.74 (95% CI: 0.70–0.79) in 1999 to 3.92 (95% CI: 3.85–4.00) in 2024, with an AAPC of 6.32 (95% CI: 5.65–7.00; *p* value < 0.001) (Table 1) (Fig. 1) (Supplemental Table 3).

### Peripheral Artery Disease with Hyperlipidemia-Related AAMR Stratified by Sex

AAMR for both men and women increased from 1999 to 2024. Among men, the AAMR increased from 0.90 (95% CI: 0.83 to 0.98) in 1999 to 4.93 (95% CI: 4.8–5.06) in 2024. Similarly, among women, the AAMR increased from 0.66 (95% CI: 0.61–0.71) in 1999 to 3.13 (95% CI: 3.04–3.22) in 2024.

Over the span of the study period, adult men exhibited slightly higher mean AAMRs compared to adult women (mean AAMR for men: 3.11, 95% CI: 2.99–3.22; for women: 1.89, 95% CI: 1.82–1.97). On average, the AAMR of both men and women increased from 1999 to 2024, with men exhibiting a significantly greater magnitude of increment than women [men: AAPC 6.40 (95% CI: 5.72–7.29; *p* < 0.001); women: AAPC 4.89 (95% CI: 4.39–5.69; *p* < 0.001)] (Table 1) (Fig. 1) (Supplemental Table 3).

### Peripheral artery disease with hyperlipidemia-related AAMR stratified by race/ethnicity

The AAMR increased across all racial/ethnic groups from 1999 to 2024: Hispanic (0.65–3.75); NH Asian or Pacific Islander (0.51–2.28); NH White (0.75–4.02); and NH Black (0.76–4.58).

The highest mean AAMRs were recorded among NH Blacks with only slight differences different among remaining racial/ethnic groups which included NH Whites, Hispanic, and NH Asian or Pacific Islander (mean AAMR: NH Black: 2.64, 95% CI: 2.41–2.86; NH White: 2.47, 95% CI: 2.39–2.54; Hispanic: 2.09, 95% CI: 1.87–2.32; NH Asian or Pacific Islander: 1.53, 95% CI: 1.27–1.82).

**Fig. 2:**
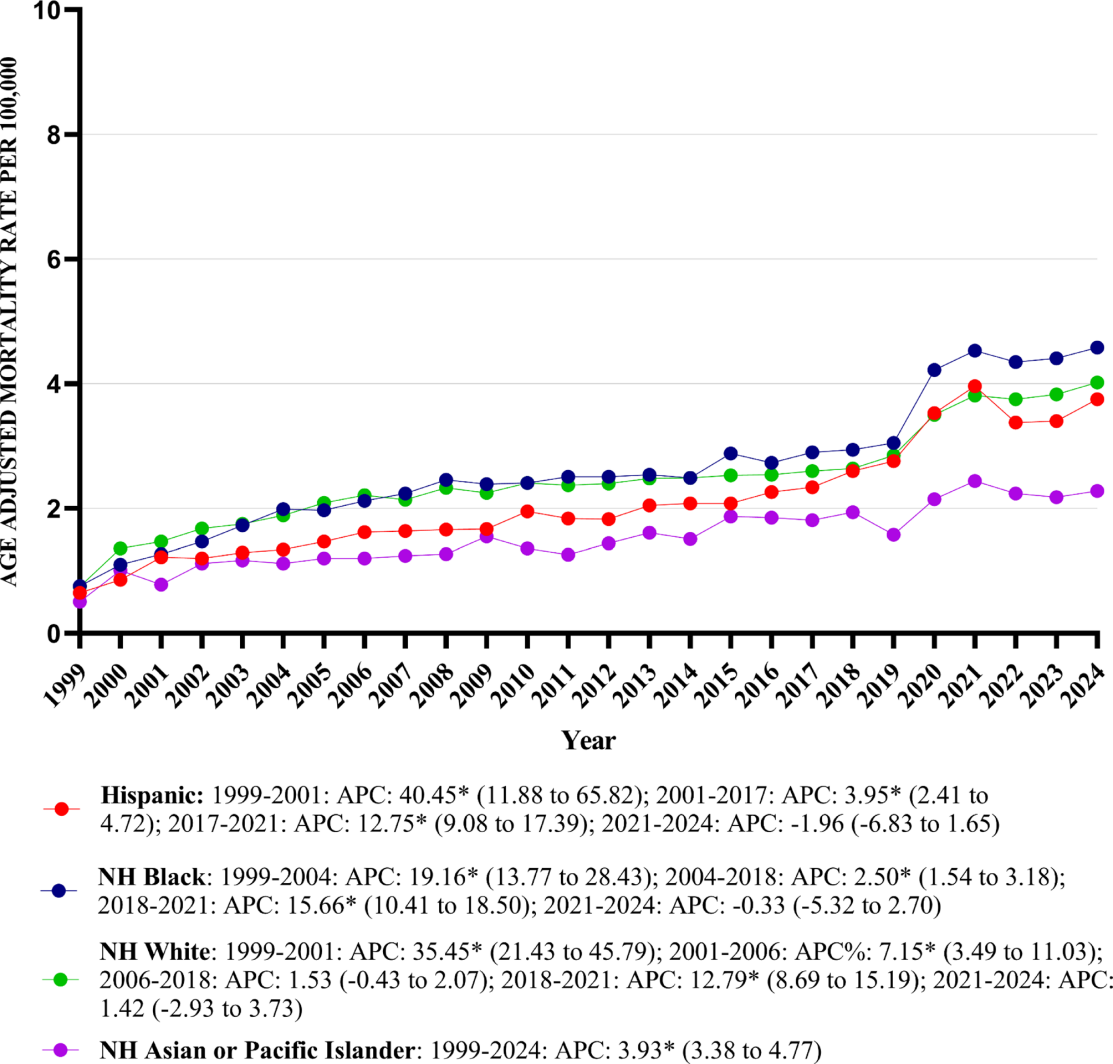
Peripheral artery disease with hyperlipidemia AAMR stratified by race per 100,000 population.

The AAMR trend of all the races increased from 1999 till 2024 [Hispanic: AAPC: 7.12, (95% CI: 5.92 to 8.52; p value < 0.001); NH Black: AAPC: 6.82, (95% CI: 6.20–7.67; *p* value < 0.001); NH White: AAPC: 6.34, (95% CI: 5.68 to 6.90; *p* value < 0.001); NH Asian or Pacific Islander: AAPC: 3.93, (95% Cl: 3.38 to 4.77; *p* value < 0.001) (Table 1) (Fig. 2) (Supplemental Table 4).

### Peripheral artery disease with hyperlipidemia-related AAMR stratified by geographical regions

#### Stratified by census region

The AAMR increased across all census regions from 1999 to 2024. In the Northeast, the AAMR increased from 0.62 in 1999 to 3.35 in 2024; in the Midwest, from 0.80 to 3.58; in the South, from 0.80 to 4.08; and in the West, from 0.79 to 4.51.

The highest mean AAMRs were observed in the West (mean AAMR: 2.93; 95% CI: 2.78–3.08), followed by the Midwest (mean AAMR: 2.61; 95% CI: 2.47–2.75), South (mean AAMR: 2.27; 95% CI: 2.16–2.37) and Northeast regions (mean AAMR: 1.93; 95% CI: 1.80–2.06).

**Fig. 3:**
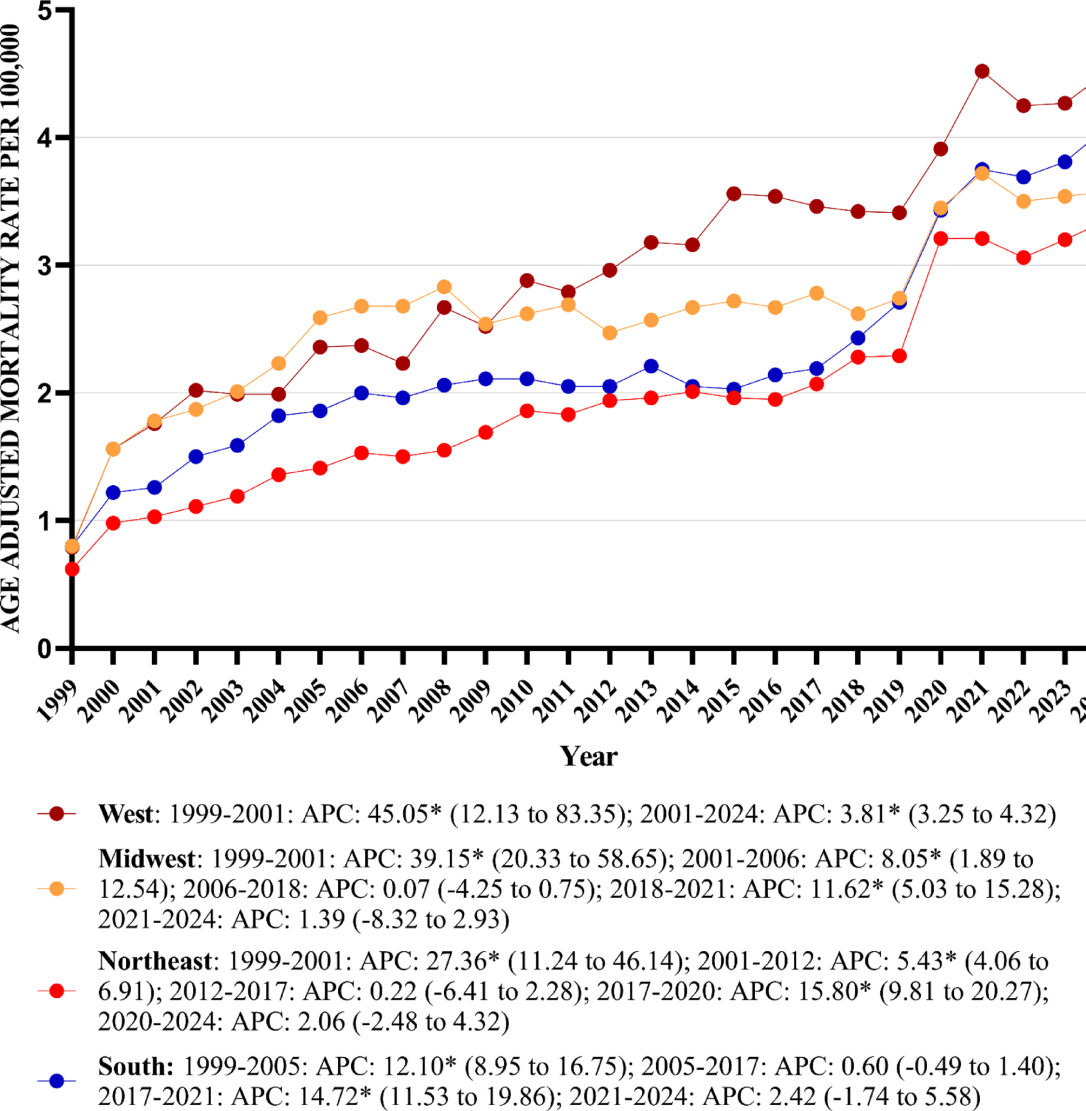
Peripheral artery disease with hyperlipidemia AAMR stratified by census per 100,000 population.

The AAMR trend of all the regions increased steadily between 1999 and 2024 [West: AAPC: 6.62, (95% CI: 5.35–8.65; *p* value < 0.001); Northeast: AAPC: 6.50, (95% CI: 5.78–7.62; *p* value < 0.001); South: AAPC: 5.67, (95% CI: 5.23–6.32; *p* value < 0.001); Midwest: AAPC: 5.45, (95% CI: 4.61–6.45; *p* value < 0.001) (Table 1) (Fig. 3) (Supplemental Table 5).

#### Stratified by urbanization

The AAMR exhibited rise in both urban and rural areas from 1999 to 2020. In urban areas, the AAMR increased from 0.73 in 1999 to 3.44 in 2020, while in rural areas it increased from 0.82 to 3.99.

Rural areas showed slightly higher mean AAMRs throughout the study period, with a mean AAMR of 2.41 for Rural (95% CI: 2.26–2.56) and 2.12 for Urban (95% CI: 2.06–2.19).

**Fig. 4:**
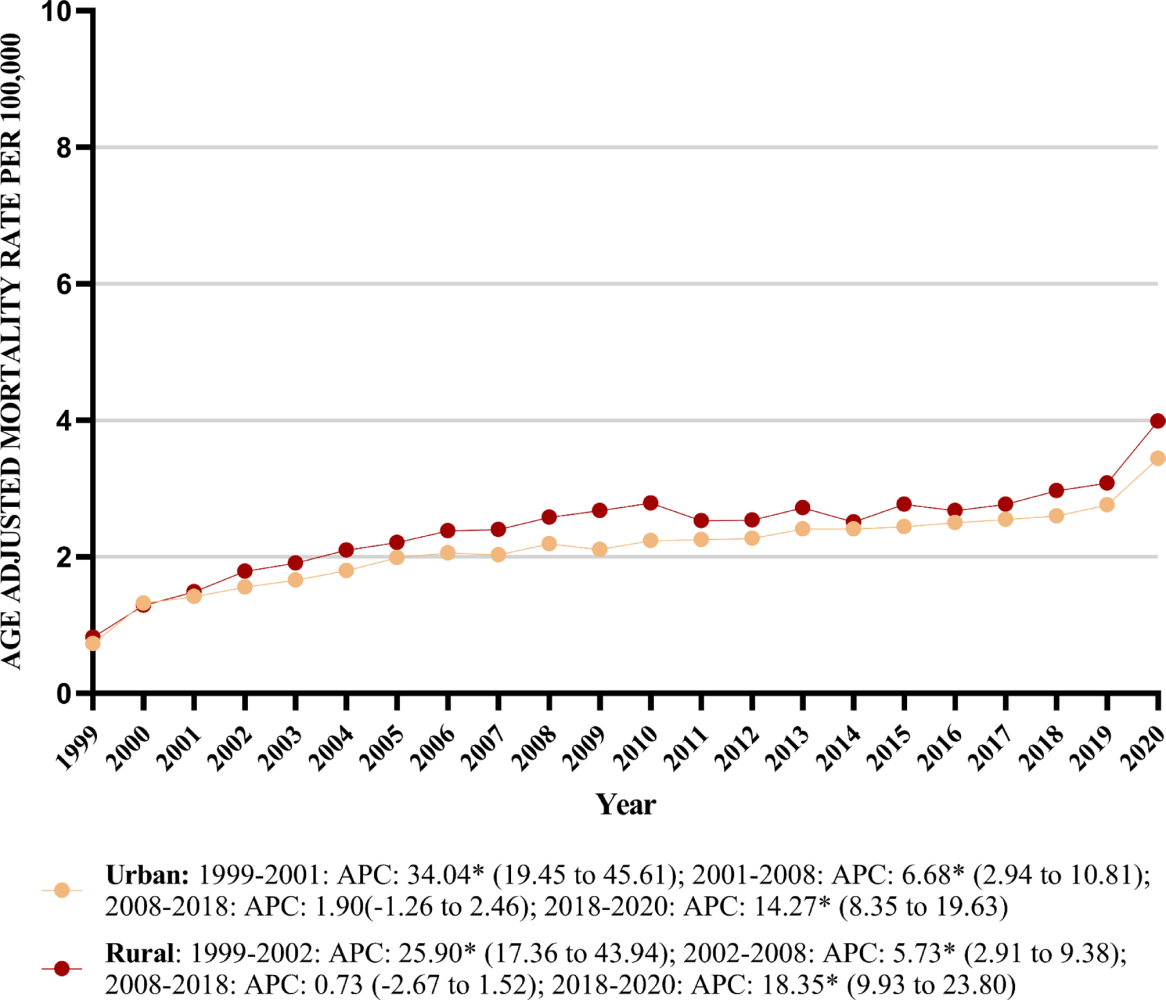
Peripheral artery disease with hyperlipidemia AAMR stratified by urbanization per 100,000 population.

The AAMR trend of both rural and urban areas increased from 1999 to 2020 with the increase more pronounced in rural areas [(Rural: AAPC: 7.07, (95% CI: 6.35–8.12; *p* value < 0.001) (Urban: AAPC: 6.90, (95% CI: 6.06–7.64; *p* value < 0.001)] (Table 1) (Fig. 4) (Supplemental Table 6).

#### Stratified by states

**Fig. 5:**
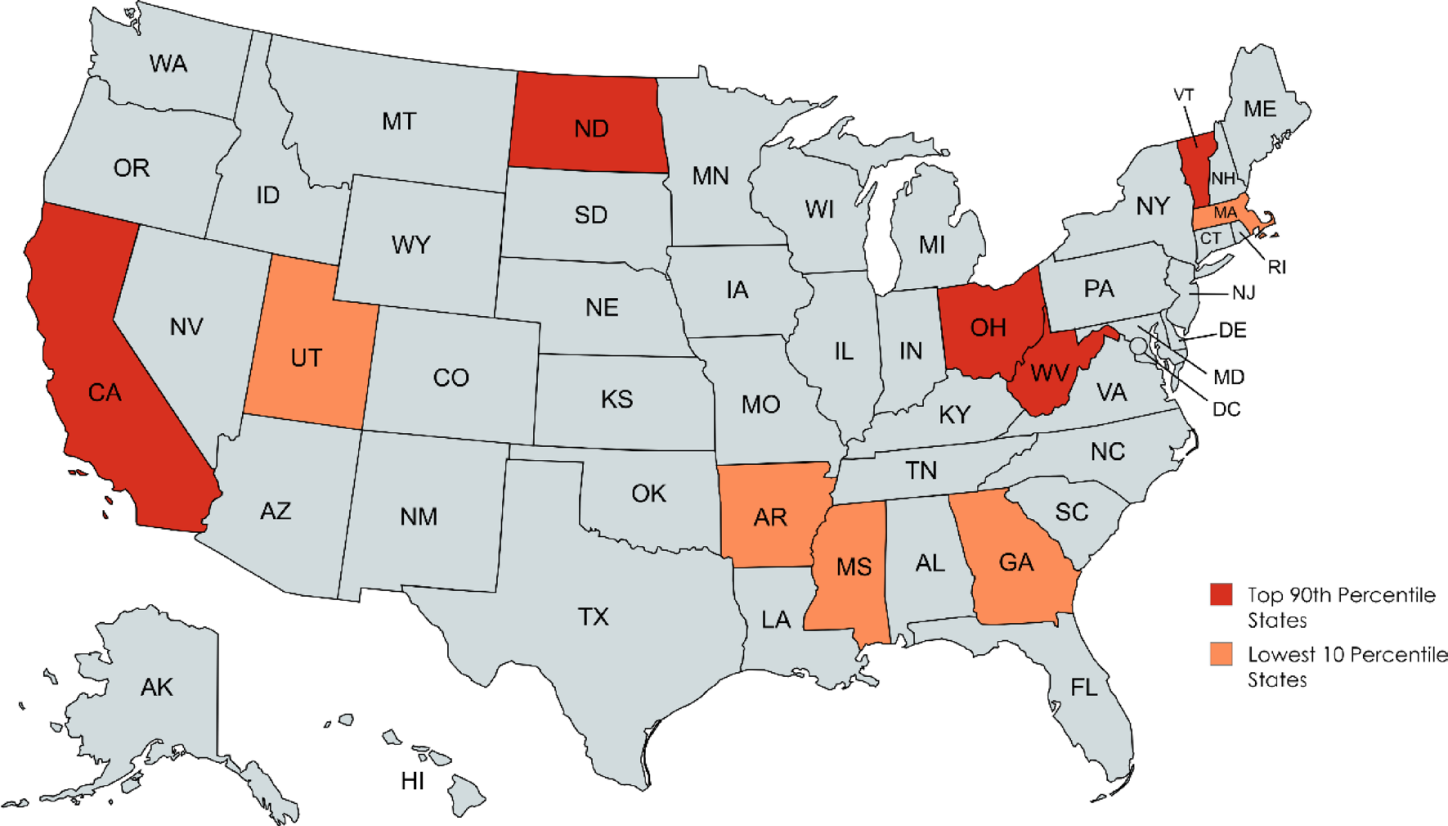
Peripheral artery disease with hyperlipidemia AAMr stratified by state per 100,000 population.

Disparities in AAMRs were manifested among different states, with AAMRs ranging from as low as 0.92 (95% CI: 0.86–0.97) in Georgia to 5.06 (95% CI: 4.63–5.49) in Vermont. States falling within the top 90th percentile included Vermont, Ohio, North Dakota, West Virginia, and California which had approximately twice higher AAMRs compared to states in the lower 10th percentile which included Georgia, Arkansas, Massachusetts, Mississippi, and Utah (Fig. 5) (Supplemental Table 7).

## Discussion

**Fig. 6:**
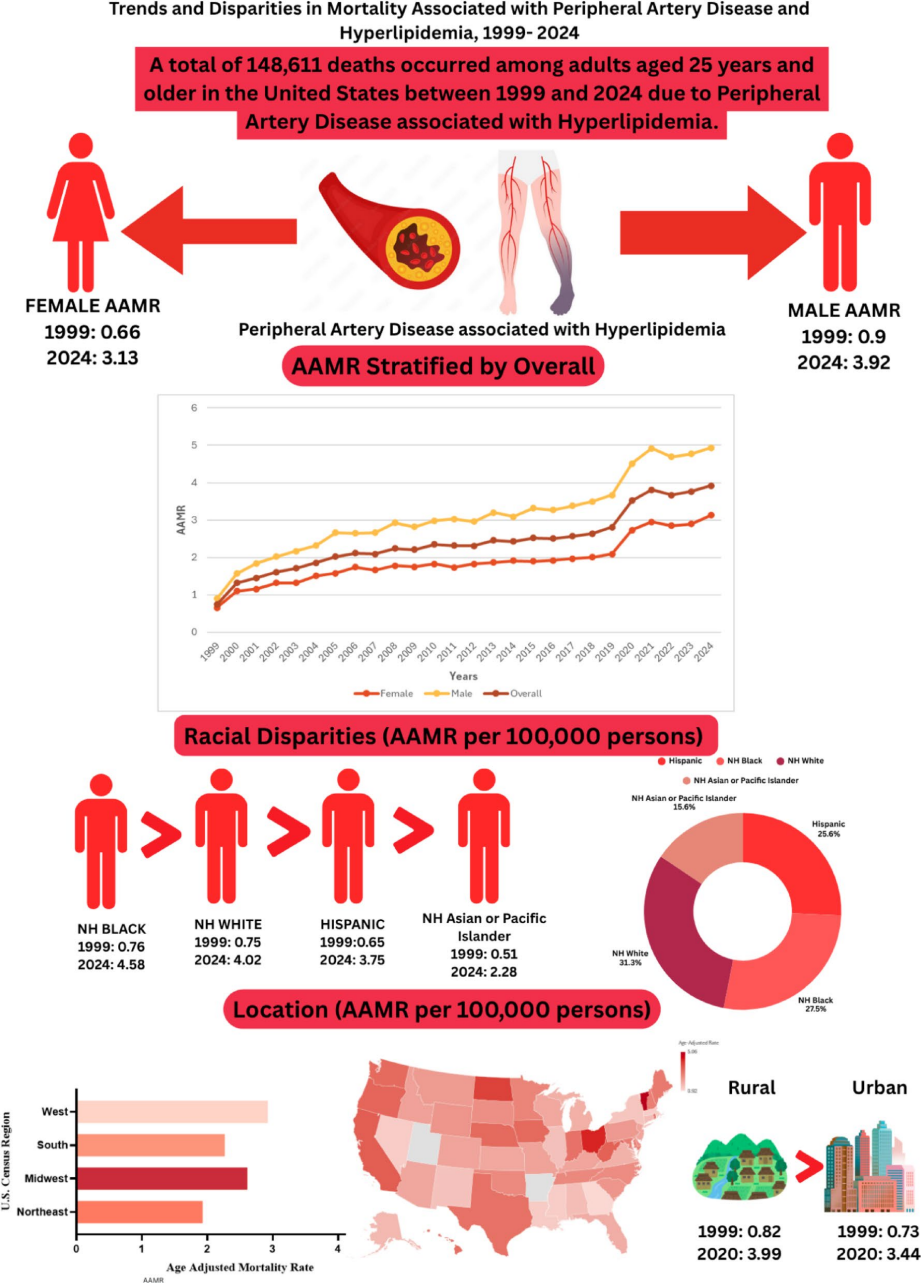
Central illustration.

The nationwide analysis of PAD with hyperlipidemia among U.S. adults over 25, based on CDC WONDER data, revealed dynamic trends and marked fluctuations in mortality. Overall mortality varied notably across the study period, with significant geographic and demographic disparities. Most deaths occurred at home, followed by medical or long-term care facilities. Men consistently showed higher mortality than women, while NH Blacks and residents of the Western region had greater mortality burdens (Fig. 6). States such as Vermont, Ohio, North Dakota, West Virginia, and California showed higher mortality compared with Georgia, Arkansas, Massachusetts, Mississippi, and Utah, highlighting pronounced geographic inequality. Rural populations also experienced slightly higher mortality, reflecting ongoing disparities in health care access and emphasizing the need for targeted interventions to reduce this burden.

PAD is a progressive narrowing or blockage of peripheral vessels by atherosclerotic plaques, resulting in reduced blood flow, intermittent claudication, poor wound healing, and, in severe cases, critical limb ischemia^17^. Hyperlipidemia may promote plaque formation through endothelial injury and cholesterol deposition^18^. Additionally, patients with PAD have a higher risk of future cardiovascular complications^19^; therefore, controlling risk factors such as hyperlipidemia, smoking, and diet is crucial for prevention and disease management^19,20^. Many deaths among patients with PAD and hyperlipidemia occurred at home, suggesting late-stage complications, diagnostic challenges^21^, or a preference for home care^22^. Deaths in medical facilities, nursing homes, and hospices indicate that PAD is often not recognized as a terminal illness unless severe. These findings highlight the need for early diagnosis, better disease management, and improved access to supportive care.

Peripheral artery disease with hyperlipidemia is a common cause of death worldwide^23,24^. In our study, overall mortality rates significantly increased throughout the study period, showing notable fluctuations driven by multiple interacting factors. Early in the observation period, a sharp rise between 1999 and 2001 was likely influenced by the growing prevalence of cardiovascular risk factors such as obesity, diabetes, and hyperlipidemia, as well as limited awareness and diagnostic capacity for PAD and lipid disorders^25–27^. However, this rapid increase may also reflect improvements in diagnostic accuracy, reporting practices, and data collection during that time. Over the following years, modest progress in cardiovascular risk management through expanded use of antihypertensive drugs, smoking cessation efforts, and better primary care screening appeared to stabilize mortality trends between 2006 and 2018^28–31^. Later, from 2018 to 2021, mortality spiked again, likely reflecting the combined effects of an aging population with accumulated vascular burden^32^, rising obesity prevalence^33^, persistent disparities in healthcare access, and the added burden of the COVID-19 pandemic^34^. More recently, mortality plateaued after 2021, possibly due to improvements in clinical recognition of PAD, the implementation of national public health strategies^35^, and updated cardiovascular health guidelines^36^.

Men consistently showed higher mortality rates compared to women. The higher mortality among men can be explained by the increased prevalence of comorbidities, including diabetes, hypertension, and hyperlipidemia, as well as a higher prevalence of smoking and greater exposure to occupational and behavioral cardiovascular risks^37–41^, all of which can accelerate PAD progression. Moreover, men tend to develop atherosclerotic plaques earlier in life, making them more vulnerable to chronic vascular disease and the subsequent development of PAD^42^. In contrast, premenopausal women have higher estrogen levels, which exert a protective effect against chronic vascular diseases and atherosclerotic plaque formation^43^. Despite higher absolute mortality rates among men, women have shown greater increases in mortality in recent years, possibly due to an aging population, rising obesity rates, and decreased physical activity^44,45^. These sex-based differences in mortality underscore the need for sex-specific, multidimensional approaches and public health strategies to reduce the mortality burden in vulnerable groups.

Significant differences in mortality rates were observed among different ethnic and racial groups, with the highest mortality seen among NH Blacks, followed by NH Whites and other groups. This disparity may be explained by the higher prevalence of smoking, hyperlipidemia, and related complications among NH Whites compared to other racial groups^46,47^. Additionally, NH Whites have an older population, making them more vulnerable, as PAD and hyperlipidemia are chronic conditions whose incidence increases with age^48^. Moreover, NH Blacks, Hispanics, and other racial groups often have less access to health care and lower rates of disease documentation compared to NH Whites, which could also contribute to the higher recorded mortality in Whites^49,50^. These findings underscore the need for large-scale public health measures, improvements in the health care system, and enhanced disease documentation, particularly among disproportionately affected populations.

Substantial geographical heterogeneity is evident in the mortality trends of PAD with hyperlipidemia. The persistent rise mortality from PAD associated with hyperlipidemia across all U.S. regions from 1999 to 2024 highlights the complex interplay of risk factor prevalence, healthcare access, and demographic changes. The sharp increases in the early 2000s, most pronounced in the West and Northeast, likely reflect the rising rates of obesity, diabetes, and uncontrolled dyslipidemia during this period, which accelerated peripheral vascular damage^51^. The stabilization observed in the Midwest and South may be partly attributable to increased statin and antihypertensive use, alongside national initiatives promoting cardiovascular risk reduction, nationwide data support these trends, suggesting that other regions, including the Northeast and West, may similarly benefit; however, region-specific data on statin utilization are not available^52–56^. Further investigation is warranted to elucidate the impact of these interventions across different regions. However, the rise in mortality after 2017 indicates persistent regional disparities in healthcare access and preventive care delivery, particularly in the South, where obesity and smoking remain highly prevalent^57–60^. The upward trend in mortality from PAD associated with hyperlipidemia highlights significant public health challenges across both rural and urban populations. Rural communities consistently exhibited slightly higher AAMRs, reflecting enduring disparities in access to preventive healthcare and a greater burden of cardiovascular risk factors^61,62^. The initial rapid increase may reflect the growing prevalence of metabolic risk factors, while the subsequent period of stabilization likely corresponds to improvements in population-level interventions such as statin use, lifestyle modification programs, and blood pressure control^53–55^. Nevertheless, the recent increase in mortality underscores persistent gaps in risk management and preventive care delivery^61^. Despite being neighboring states, Massachusetts had one of the lowest AAMRs, whereas Rhode Island’s rate was comparatively higher. Such discrepancies may stem from differences in population size, socioeconomic characteristics, healthcare access, and the prevalence of cardiovascular risk factors. Variability in data reporting practices across states may also contribute. Moreover, Massachusetts’ long-standing public health initiatives, preventive screening programs, and widespread statin use may help explain its relatively lower mortality burden^63^.

Overall, the disproportionate rise in PAD with hyperlipidemia-related mortality between 2018 and 2021 coincided with the COVID-19 pandemic. Limited access to vascular care, including delays in routine surveillance, elective interventions, and timely revascularization, likely contributed to disease progression and more advanced ischemic presentations^64^. Additionally, SARS-CoV-2 infection has been associated with endothelial dysfunction, systemic inflammation, and a prothrombotic state, all of which can accelerate atherosclerotic progression and precipitate acute ischemic events^65,66^. These observations align with prior studies reporting worse outcomes in PAD patients treated during the pandemic compared to pre-pandemic cohorts^67^. Beyond the acute effects described previously, SARS-CoV-2 may exert synergistic vascular damage in patients with hyperlipidemia and PAD by sustaining low-grade endothelial inflammation and impairing vascular repair mechanisms, thereby accelerating atherosclerotic plaque progression^68^. Furthermore, the disruptions in outpatient follow-up, preventive care, and medication management during COVID-19 lockdowns further reduced adherence to lipid-lowering and antiplatelet therapies, predisposing patients to worsening atherosclerotic burden^69^.Together, these findings highlighted the need for timely intervention and extended follow-ups for PAD patients, particularly during global health crises, to mitigate the compounded risk imposed by both disease progression and pandemic-related limitations in care.

The future management of PAD in hyperlipidemic populations requires a systems-based approach that combines therapeutic innovation with structural reforms. Next-generation lipid-lowering therapies such as PCSK9 inhibitors and bempedoic acid represent important pharmaceutical advances for aggressive lipid control^70,71^. Incorporating dual antithrombotic therapy into standard protocols has the potential to reduce rehospitalizations and health care costs^72^. Regenerative medicines, including nanoparticles and gene editing represent a frontier for vascular repair^73,74^. Finally, embedding early diagnosis and medical management into health systems will be critical to maximizing the global reach of both digital and pharmacological innovations^75,76^.

### Limitations

This study is subject to several limitations. First, because of the dependence on ICD codes and death certificates, there is a risk of misclassification or omission of PAD with hyperlipidemia as a cause of death. Second, reliance on specific coding and documentation may lead to underreporting or missed cases of PAD with hyperlipidemia. Third, the increased use of electronic health records for diagnosing PAD may lead to potential overestimation of PAD–hyperlipidemia co-listing on death certificates, potentially skewing the perception of PAD-related mortality trends. Fourth, certain variables, such as patients’ socioeconomic status, were not evaluated due to the unavailability of relevant data. Fifth, certain variables, such as patients’ socioeconomic status, laboratory data, clinical findings, treatment histories, and disease-specific characteristics (e.g., ankle-brachial index, vascular imaging, lipid profile, or genetic data), were unavailable, limiting the depth of analysis. Sixth, co-morbid conditions, including diabetes, hypertension, coronary artery disease, end-stage renal disease, heart failure, and treatment variables may confound mortality trends. Seventh, urbanization data were not available after 2020, restricting stratified analyses beyond that year. Finally, the database lacks information on disease-specific characteristics necessary to further define PAD with hyperlipidemia, such as ankle-brachial index, vascular imaging findings, lipid panel results, or genetic testing data.

## Supplementary Information

Below is the link to the electronic supplementary material.

Supplementary Material 1

## Data Availability

The data supporting the findings of this study were obtained from the CDC WONDER online database (Centers for Disease Control and Prevention Wide-ranging Online Data for Epidemiologic Research). The datasets used and analyzed during the current study are publicly available and can be accessed at [CDC WONDER] (https://wonder.cdc.gov).
