# Supplementary material for: Trends and disparities in mortality associated with peripheral artery disease and hyperlipidemia, 1999–2024
Source: Sci Rep. 2025 Nov 22;15:45008. doi: 10.1038/s41598-025-29224-7 (PMC12748544; doi:10.1038/s41598-025-29224-7)
Supplement: Supplementary file 1 — Supplementary Material 1 [file 41598_2025_29224_MOESM1_ESM.docx]

**Supplemental Table 1, Peripheral Artery Disease related mortalities in Adults with Hyperlipidemia, Stratified by Sex and Race, in the United States, 1999 to 2024**

| **Supplemental Table 1, Peripheral Artery Disease related mortalities in Adults with Hyperlipidemia, Stratified by Sex and Race, in the United States, 1999 to 2024** | | | | | | | | |
| --- | --- | --- | --- | --- | --- | --- | --- | --- |
| **Deaths** | | | | | | | | |
| **Year** | **Overall** | **Women** | **Men** | **Non-Hispanic Asian or Pacific Islander** | **Non-Hispanic Whites** | **Non-Hispanic Blacks** | **Hispanic** | **Population** |
| **1999** | 1342 | 700 | 642 | 20 | 1152 | 112 | 53 | 180408769 |
| **2000** | 2359 | 1195 | 1164 | 44 | 2061 | 164 | 74 | 181984640 |
| **2001** | 2612 | 1255 | 1357 | 38 | 2249 | 197 | 117 | 184305128 |
| **2002** | 2963 | 1446 | 1517 | 50 | 2560 | 221 | 115 | 186208028 |
| **2003** | 3185 | 1494 | 1691 | 57 | 2708 | 269 | 133 | 188090429 |
| **2004** | 3507 | 1720 | 1787 | 57 | 2967 | 319 | 146 | 190205384 |
| **2005** | 3953 | 1840 | 2113 | 65 | 3371 | 323 | 176 | 192551384 |
| **2006** | 4201 | 2058 | 2143 | 69 | 3570 | 348 | 193 | 195019359 |
| **2007** | 4231 | 2031 | 2200 | 74 | 3550 | 382 | 196 | 197403777 |
| **2008** | 4657 | 2181 | 2476 | 79 | 3919 | 419 | 213 | 199795090 |
| **2009** | 4634 | 2209 | 2425 | 106 | 3852 | 425 | 225 | 202107016 |
| **2010** | 4972 | 2359 | 2613 | 95 | 4139 | 437 | 281 | 203891983 |
| **2011** | 5054 | 2311 | 2743 | 99 | 4160 | 472 | 285 | 206592936 |
| **2012** | 5237 | 2474 | 2763 | 117 | 4296 | 487 | 298 | 208826037 |
| **2013** | 5648 | 2552 | 3096 | 145 | 4596 | 514 | 347 | 211085314 |
| **2014** | 5749 | 2672 | 3077 | 136 | 4673 | 515 | 377 | 213809280 |
| **2015** | 6115 | 2727 | 3388 | 190 | 4836 | 619 | 398 | 216553817 |
| **2016** | 6263 | 2828 | 3435 | 201 | 4944 | 620 | 445 | 218641417 |
| **2017** | 6538 | 2929 | 3609 | 208 | 5095 | 680 | 497 | 221447331 |
| **2018** | 6954 | 3072 | 3882 | 239 | 5349 | 725 | 578 | 223311190 |
| **2019** | 7445 | 3246 | 4199 | 200 | 5809 | 754 | 627 | 224981167 |
| **2020** | 9500 | 4267 | 5233 | 292 | 7213 | 1066 | 847 | 226635013 |
| **2021** | 10038 | 4427 | 5611 | 309 | 7500 | 1133 | 961 | 228238412 |
| **2022** | 10146 | 4580 | 5566 | 308 | 7740 | 1108 | 856 | 229508599 |
| **2023** | 10375 | 4609 | 5766 | 313 | 7888 | 1151 | 893 | 231529762 |
| **2024** | 10933 | 4957 | 5976 | 324 | 8282 | 1207 | 962 | 231529762 |
| **Total** | **148611** | **68139** | **80472** | **3835** | **118479** | **14667** | **10293** | **5394661024** |

**Supplemental Table 2, Peripheral Artery Disease related Mortality, Stratified by Place of Death, in Adults with Hyperlipidemia in the United States, 1999 to 2024**

| **Supplemental Table 2, Peripheral Artery Disease related Mortality, Stratified by Place of Death, in Adults with Hyperlipidemia in the United States, 1999 to 2024** | | | | |
| --- | --- | --- | --- | --- |
| **Deaths** | | | | |
| **Year** | **Medical Facility** | **Nursing Home/Long-term Care Facility** | **Hospice Facility** | **Decedent's Home** |
| **1999** | 474 | 208 | - | 377 |
| **2000** | 793 | 390 | - | 694 |
| **2001** | 849 | 433 | - | 787 |
| **2002** | 937 | 516 | - | 904 |
| **2003** | 988 | 555 | - | 991 |
| **2004** | 1071 | 637 | - | 1068 |
| **2005** | 1109 | 765 | 48 | 1263 |
| **2006** | 1215 | 786 | 63 | 1307 |
| **2007** | 1145 | 830 | 97 | 1370 |
| **2008** | 1256 | 888 | 94 | 1525 |
| **2009** | 1215 | 912 | 76 | 1549 |
| **2010** | 1287 | 993 | 84 | 1721 |
| **2011** | 1266 | 1037 | 106 | 1743 |
| **2012** | 1274 | 1088 | 122 | 1842 |
| **2013** | 1297 | 1186 | 171 | 2056 |
| **2014** | 1228 | 1249 | 164 | 2188 |
| **2015** | 1282 | 1328 | 177 | 2344 |
| **2016** | 1212 | 1372 | 198 | 2528 |
| **2017** | 1259 | 1440 | 221 | 2623 |
| **2018** | 1356 | 1505 | 310 | 2727 |
| **2019** | 1459 | 1568 | 363 | 2908 |
| **2020** | 1768 | 2010 | 341 | 3959 |
| **2021** | 6421 | 1790 | 446 | 4399 |
| **2022** | 6184 | 1864 | 435 | 4581 |
| **2023** | 6329 | 1915 | 488 | 4597 |
| **2024** | 6804 | 2071 | 614 | 4674 |
| **Total** | **51478** | **29336** | **4618** | **56725** |

**Supplemental Table 3 Overall and Sex‐Stratified Peripheral Artery Disease related Age-Adjusted Mortality Rates per 100,000, in Adults with Hyperlipidemia in the United States, 1999 to 2024**

| **Supplemental Table 3 Overall and Sex‐Stratified Peripheral Artery Disease related Age-Adjusted Mortality Rates per 100,000, in Adults with Hyperlipidemia in the United States, 1999 to 2024** | | | |
| --- | --- | --- | --- |
| **Age-Adjusted Rate (95% CI)** | | | |
| **Year** | **Men** | **Women** | **Overall** |
| **1999** | 0.90 (0.83 - 0.98) | 0.66 (0.61 - 0.71) | 0.74 (0.70 - 0.79) |
| **2000** | 1.57 (1.48 - 1.66) | 1.10 (1.04 - 1.16) | 1.32 (1.27 - 1.38) |
| **2001** | 1.84 (1.74 - 1.94) | 1.16 (1.10 - 1.23) | 1.45 (1.40 - 1.51) |
| **2002** | 2.02 (1.92 - 2.13) | 1.32 (1.25 - 1.38) | 1.61 (1.56 - 1.67) |
| **2003** | 2.17 (2.07 - 2.28) | 1.32 (1.25 - 1.39) | 1.71 (1.65 - 1.77) |
| **2004** | 2.32 (2.21 - 2.42) | 1.51 (1.43 - 1.58) | 1.86 (1.79 - 1.92) |
| **2005** | 2.66 (2.54 - 2.77) | 1.58 (1.51 - 1.66) | 2.02 (1.96 - 2.09) |
| **2006** | 2.65 (2.54 - 2.77) | 1.74 (1.67 - 1.82) | 2.12 (2.05 - 2.18) |
| **2007** | 2.66 (2.55 - 2.78) | 1.66 (1.59 - 1.73) | 2.09 (2.03 - 2.15) |
| **2008** | 2.93 (2.81 - 3.05) | 1.78 (1.71 - 1.86) | 2.24 (2.18 - 2.31) |
| **2009** | 2.82 (2.71 - 2.94) | 1.75 (1.68 - 1.83) | 2.21 (2.14 - 2.27) |
| **2010** | 2.98 (2.86 - 3.10) | 1.83 (1.75 - 1.90) | 2.35 (2.28 - 2.41) |
| **2011** | 3.03 (2.92 - 3.15) | 1.73 (1.66 - 1.80) | 2.32 (2.25 - 2.38) |
| **2012** | 2.96 (2.85 - 3.08) | 1.83 (1.76 - 1.90) | 2.31 (2.25 - 2.38) |
| **2013** | 3.20 (3.09 - 3.32) | 1.87 (1.79 - 1.94) | 2.46 (2.40 - 2.53) |
| **2014** | 3.09 (2.98 - 3.20) | 1.91 (1.83 - 1.98) | 2.43 (2.36 - 2.49) |
| **2015** | 3.32 (3.21 - 3.44) | 1.90 (1.83 - 1.97) | 2.52 (2.45 - 2.58) |
| **2016** | 3.27 (3.16 - 3.38) | 1.92 (1.84 - 1.99) | 2.51 (2.45 - 2.57) |
| **2017** | 3.38 (3.27 - 3.49) | 1.97 (1.89 - 2.04) | 2.57 (2.51 - 2.63) |
| **2018** | 3.50 (3.39 - 3.62) | 2.01 (1.93 - 2.08) | 2.64 (2.58 - 2.71) |
| **2019** | 3.67 (3.56 - 3.78) | 2.09 (2.02 - 2.17) | 2.81 (2.74 - 2.87) |
| **2020** | 4.51 (4.38 - 4.63) | 2.73 (2.65 - 2.81) | 3.52 (3.45 - 3.59) |
| **2021** | 4.92 (4.78 - 5.05) | 2.95 (2.86 - 3.04) | 3.81 (3.73 - 3.88) |
| **2022** | 4.69 (4.56 - 4.81) | 2.85 (2.77 - 2.94) | 3.67 (3.60 - 3.74) |
| **2023** | 4.77 (4.65 - 4.90) | 2.90 (2.82 - 2.99) | 3.76 (3.68 - 3.83) |
| **2024** | 4.93 (4.80 - 5.06) | 3.13 (3.04 - 3.22) | 3.92 (3.85 - 4.00) |
| **Total** | **3.11 (2.99 - 3.22)** | **1.89 (1.82 - 1.97)** | **2.42 (2.36 - 2.49)** |

**Supplemental Table 4 Race‐Stratified Peripheral Artery Disease related Age-Adjusted Mortality Rates per 100,000 in Adults with Hyperlipidemia in the United States, 1999 to 2024**

| **Supplemental Table 4 Race‐Stratified Peripheral Artery Disease related Age-Adjusted Mortality Rates per 100,000 in Adults with Hyperlipidemia in the United States, 1999 to 2024** | | | | |
| --- | --- | --- | --- | --- |
| **Age-Adjusted Rate (95% CI)** | | | | |
| **Year** | **Non-Hispanic Asian or Pacific Islander** | **Non-Hispanic Black** | **Hispanic** | **Non-Hispanic White** |
| **1999** | 0.51 (0.31 - 0.8) | 0.76 (0.61 - 0.9) | 0.65 (0.48 - 0.86) | 0.75 (0.71 - 0.8) |
| **2000** | 1.01 (0.72 - 1.38) | 1.1 (0.93 - 1.27) | 0.86 (0.67 - 1.08) | 1.36 (1.3 - 1.42) |
| **2001** | 0.78 (0.54 - 1.08) | 1.27 (1.09 - 1.45) | 1.22 (0.99 - 1.45) | 1.47 (1.41 - 1.53) |
| **2002** | 1.12 (0.83 - 1.49) | 1.47 (1.27 - 1.66) | 1.2 (0.97 - 1.43) | 1.68 (1.62 - 1.75) |
| **2003** | 1.17 (0.88 - 1.53) | 1.73 (1.52 - 1.95) | 1.29 (1.07 - 1.52) | 1.75 (1.68 - 1.82) |
| **2004** | 1.12 (0.84 - 1.47) | 1.99 (1.77 - 2.21) | 1.34 (1.12 - 1.57) | 1.89 (1.83 - 1.96) |
| **2005** | 1.2 (0.92 - 1.54) | 1.97 (1.75 - 2.19) | 1.47 (1.25 - 1.69) | 2.09 (2.02 - 2.16) |
| **2006** | 1.2 (0.93 - 1.53) | 2.12 (1.89 - 2.34) | 1.62 (1.38 - 1.86) | 2.21 (2.14 - 2.29) |
| **2007** | 1.24 (0.97 - 1.56) | 2.24 (2.01 - 2.47) | 1.64 (1.4 - 1.87) | 2.14 (2.07 - 2.22) |
| **2008** | 1.27 (1.1 - 1.58) | 2.46 (2.22 - 2.7) | 1.66 (1.43 - 1.89) | 2.33 (2.25 - 2.4) |
| **2009** | 1.55 (1.25 - 1.85) | 2.39 (2.16 - 2.63) | 1.67 (1.45 - 1.9) | 2.25 (2.18 - 2.32) |
| **2010** | 1.36 (1.09 - 1.67) | 2.41 (2.18 - 2.64) | 1.95 (1.71 - 2.18) | 2.41 (2.33 - 2.48) |
| **2011** | 1.26 (1.02 - 1.55) | 2.51 (2.28 - 2.75) | 1.84 (1.62 - 2.06) | 2.37 (2.3 - 2.44) |
| **2012** | 1.44 (1.18 - 1.71) | 2.51 (2.28 - 2.74) | 1.83 (1.61 - 2.04) | 2.4 (2.33 - 2.47) |
| **2013** | 1.61 (1.34 - 1.87) | 2.54 (2.32 - 2.77) | 2.05 (1.83 - 2.27) | 2.48 (2.41 - 2.55) |
| **2014** | 1.51 (1.25 - 1.76) | 2.49 (2.27 - 2.71) | 2.08 (1.86 - 2.29) | 2.49 (2.41 - 2.56) |
| **2015** | 1.87 (1.6 - 2.14) | 2.88 (2.65 - 3.12) | 2.08 (1.87 - 2.29) | 2.53 (2.45 - 2.6) |
| **2016** | 1.85 (1.59 - 2.11) | 2.73 (2.51 - 2.95) | 2.26 (2.04 - 2.47) | 2.54 (2.47 - 2.61) |
| **2017** | 1.81 (1.56 - 2.05) | 2.9 (2.68 - 3.13) | 2.34 (2.13 - 2.56) | 2.6 (2.53 - 2.68) |
| **2018** | 1.94 (1.69 - 2.19) | 2.94 (2.72 - 3.16) | 2.6 (2.38 - 2.81) | 2.64 (2.56 - 2.71) |
| **2019** | 1.58 (1.36 - 1.8) | 3.05 (2.83 - 3.27) | 2.76 (2.54 - 2.98) | 2.85 (2.77 - 2.92) |
| **2020** | 2.15 (1.9 - 2.4) | 4.22 (3.96 - 4.48) | 3.53 (3.29 - 3.77) | 3.5 (3.42 - 3.58) |
| **2021** | 2.44 (2.16 - 2.71) | 4.53 (4.25 - 4.8) | 3.96 (3.7 - 4.22) | 3.81 (3.72 - 3.9) |
| **2022** | 2.24 (1.99 - 2.5) | 4.35 (4.09 - 4.61) | 3.38 (3.15 - 3.61) | 3.75 (3.66 - 3.83) |
| **2023** | 2.18 (1.94 - 2.42) | 4.41 (4.15 - 4.68) | 3.4 (3.17 - 3.63) | 3.83 (3.75 - 3.92) |
| **2024** | 2.28 (2.03 - 2.53) | 4.58 (4.31 - 4.84) | 3.75 (3.51 - 3.99) | 4.02 (3.93 - 4.1) |
| **Total** | **1.53 (1.27 - 1.82)** | **2.64 (2.41 - 2.86)** | **2.09 (1.87 - 2.32)** | **2.47 (2.39 - 2.54)** |
| NH = Non-Hispanic | | | | |

**Supplemental Table 5, Peripheral Artery Disease related Age-Adjusted Mortality Rates per 100,000, Stratified by Census Region, in Adults with Hyperlipidemia in the United States, 1999 to 2024**

| **Supplemental Table 5, Peripheral Artery Disease related Age-Adjusted Mortality Rates per 100,000, Stratified by Census Region, in Adults with Hyperlipidemia in the United States, 1999 to 2024** | | | | |
| --- | --- | --- | --- | --- |
|  | **Census Region: Northeast** | **Census Region: Midwest** | **Census Region: South** | **Census Region: West** |
| **Year** | **Age-Adjusted Rate (95% CI)** | **Age-Adjusted Rate (95% CI)** | **Age-Adjusted Rate (95% CI)** | **Age-Adjusted Rate (95% CI)** |
| **1999** | 0.62 (0.54 - 0.70) | 0.80 (0.72 - 0.89) | 0.80 (0.73 - 0.87) | 0.79 (0.70 - 0.89) |
| **2000** | 0.98 (0.88 - 1.08) | 1.56 (1.44 - 1.68) | 1.22 (1.13 - 1.31) | 1.56 (1.43 - 1.69) |
| **2001** | 1.03 (0.92 - 1.13) | 1.78 (1.65 - 1.90) | 1.26 (1.17 - 1.35) | 1.76 (1.62 - 1.90) |
| **2002** | 1.11 (1.00 - 1.21) | 1.87 (1.74 - 2.00) | 1.50 (1.40 - 1.60) | 2.02 (1.88 - 2.17) |
| **2003** | 1.19 (1.08 - 1.30) | 2.01 (1.88 - 2.14) | 1.59 (1.50 - 1.69) | 1.99 (1.85 - 2.13) |
| **2004** | 1.36 (1.24 - 1.47) | 2.23 (2.09 - 2.37) | 1.82 (1.71 - 1.92) | 1.99 (1.85 - 2.14) |
| **2005** | 1.41 (1.29 - 1.53) | 2.59 (2.44 - 2.74) | 1.86 (1.76 - 1.97) | 2.36 (2.21 - 2.51) |
| **2006** | 1.53 (1.41 - 1.65) | 2.68 (2.53 - 2.83) | 2.00 (1.90 - 2.11) | 2.37 (2.22 - 2.52) |
| **2007** | 1.50 (1.38 - 1.62) | 2.68 (2.53 - 2.83) | 1.96 (1.86 - 2.06) | 2.23 (2.09 - 2.38) |
| **2008** | 1.55 (1.43 - 1.67) | 2.83 (2.68 - 2.98) | 2.06 (1.95 - 2.16) | 2.67 (2.52 - 2.83) |
| **2009** | 1.69 (1.56 - 1.81) | 2.54 (2.40 - 2.69) | 2.11 (2.01 - 2.22) | 2.52 (2.37 - 2.67) |
| **2010** | 1.86 (1.73 - 1.99) | 2.62 (2.47 - 2.76) | 2.11 (2.01 - 2.21) | 2.88 (2.72 - 3.03) |
| **2011** | 1.83 (1.71 - 1.96) | 2.69 (2.54 - 2.83) | 2.05 (1.95 - 2.15) | 2.79 (2.64 - 2.94) |
| **2012** | 1.94 (1.81 - 2.08) | 2.47 (2.33 - 2.61) | 2.05 (1.95 - 2.15) | 2.96 (2.80 - 3.11) |
| **2013** | 1.96 (1.83 - 2.09) | 2.57 (2.43 - 2.71) | 2.21 (2.11 - 2.31) | 3.18 (3.02 - 3.34) |
| **2014** | 2.01 (1.88 - 2.14) | 2.67 (2.53 - 2.82) | 2.05 (1.95 - 2.15) | 3.16 (3.00 - 3.31) |
| **2015** | 1.96 (1.83 - 2.09) | 2.72 (2.58 - 2.86) | 2.03 (1.93 - 2.12) | 3.56 (3.40 - 3.72) |
| **2016** | 1.95 (1.82 - 2.08) | 2.67 (2.53 - 2.81) | 2.14 (2.04 - 2.24) | 3.54 (3.38 - 3.70) |
| **2017** | 2.07 (1.94 - 2.20) | 2.78 (2.64 - 2.92) | 2.19 (2.09 - 2.28) | 3.46 (3.30 - 3.61) |
| **2018** | 2.28 (2.14 - 2.41) | 2.62 (2.48 - 2.75) | 2.43 (2.33 - 2.53) | 3.42 (3.27 - 3.58) |
| **2019** | 2.29 (2.15 - 2.42) | 2.74 (2.60 - 2.87) | 2.71 (2.60 - 2.81) | 3.41 (3.26 - 3.57) |
| **2020** | 3.21 (3.06 - 3.37) | 3.45 (3.30 - 3.61) | 3.43 (3.31 - 3.54) | 3.91 (3.75 - 4.07) |
| **2021** | 3.21 (3.06 - 3.37) | 3.72 (3.55 - 3.88) | 3.75 (3.63 - 3.87) | 4.52 (4.35 - 4.70) |
| **2022** | 3.06 (2.91 - 3.21) | 3.50 (3.35 - 3.65) | 3.69 (3.58 - 3.81) | 4.25 (4.09 - 4.42) |
| **2023** | 3.20 (3.04 - 3.35) | 3.54 (3.39 - 3.70) | 3.81 (3.69 - 3.92) | 4.27 (4.11 - 4.43) |
| **2024** | 3.35 (3.19 - 3.50) | 3.58 (3.43 - 3.74) | 4.08 (3.96 - 4.20) | 4.51 (4.35 - 4.68) |
| **Total** | **1.93 (1.80 - 2.06)** | **2.61 (2.47 - 2.75)** | **2.27 (2.16 - 2.37)** | **2.93 (2.78 - 3.08)** |

**Supplemental Table 6, Peripheral Artery Disease related Age-Adjusted Mortality Rates per 100,000, Stratified by Urban-Rural Classification, in Adults with Hyperlipidemia in the United States, 1999 to 2020**

| **Supplemental Table 6, Peripheral Artery Disease related Age-Adjusted Mortality Rates per 100,000, Stratified by Urban-Rural Classification, in Adults with Hyperlipidemia in the United States, 1999 to 2020** | | |
| --- | --- | --- |
| **Age-Adjusted Rate (95% CI)** | | |
| **Year** | **Urban** | **Rural** |
| **1999** | 0.73 (0.69 - 0.78) | 0.82 (0.72 - 0.92) |
| **2000** | 1.32 (1.26 - 1.38) | 1.29 (1.17 - 1.41) |
| **2001** | 1.42 (1.36 - 1.48) | 1.49 (1.36 - 1.62) |
| **2002** | 1.56 (1.49 - 1.62) | 1.79 (1.65 - 1.93) |
| **2003** | 1.66 (1.59 - 1.72) | 1.91 (1.76 - 2.06) |
| **2004** | 1.8 (1.73 - 1.86) | 2.1 (1.95 - 2.25) |
| **2005** | 1.99 (1.92 - 2.06) | 2.21 (2.06 - 2.37) |
| **2006** | 2.06 (1.99 - 2.13) | 2.38 (2.22 - 2.54) |
| **2007** | 2.03 (1.96 - 2.1) | 2.4 (2.24 - 2.56) |
| **2008** | 2.19 (2.12 - 2.26) | 2.58 (2.42 - 2.75) |
| **2009** | 2.11 (2.04 - 2.18) | 2.68 (2.52 - 2.85) |
| **2010** | 2.24 (2.17 - 2.31) | 2.79 (2.62 - 2.96) |
| **2011** | 2.25 (2.18 - 2.32) | 2.53 (2.37 - 2.69) |
| **2012** | 2.27 (2.2 - 2.34) | 2.54 (2.39 - 2.7) |
| **2013** | 2.41 (2.34 - 2.48) | 2.72 (2.56 - 2.88) |
| **2014** | 2.41 (2.34 - 2.48) | 2.51 (2.35 - 2.66) |
| **2015** | 2.44 (2.37 - 2.51) | 2.77 (2.61 - 2.93) |
| **2016** | 2.5 (2.43 - 2.57) | 2.68 (2.52 - 2.84) |
| **2017** | 2.55 (2.48 - 2.62) | 2.77 (2.61 - 2.93) |
| **2018** | 2.6 (2.53 - 2.66) | 2.97 (2.81 - 3.14) |
| **2019** | 2.76 (2.69 - 2.83) | 3.08 (2.91 - 3.25) |
| **2020** | 3.44 (3.36 - 3.52) | 3.99 (3.8 - 4.18) |
| **Total** | **2.12 (2.06 - 2.19)** | **2.41 (2.26 - 2.56)** |
| The data for urbanization is only available till 2020 in the CDC Wonder Database. | | |
|  |  |  |

**Supplemental Table 7, Peripheral Artery Disease related Age-Adjusted Mortality Rates per 100,000, Stratified by States, in Adults with Hyperlipidemia in the United States, 1999 to 2024**

| **Supplemental Table 7, Peripheral Artery Disease related Age-Adjusted Mortality Rates per 100,000, Stratified by States, in Adults with Hyperlipidemia in the United States, 1999 to 2024** | |
| --- | --- |
| **State** | **Age-Adjusted Rate (95% CI)** |
| Alabama | 1.45 (1.36 - 1.53) |
| Alaska | 1.55 (1.24 - 1.92) |
| Arizona | 1.90 (1.82 - 1.99) |
| Arkansas | 1.09 (1.00 - 1.19) |
| California | 3.33 (3.28 - 3.38) |
| Colorado | 1.96 (1.85 - 2.07) |
| Connecticut | 1.48 (1.39 - 1.58) |
| Delaware | 2.36 (2.11 - 2.61) |
| District of Columbia | 1.36 (1.11 - 1.61) |
| Florida | 1.77 (1.72 - 1.81) |
| Georgia | 0.92 (0.86 - 0.97) |
| Hawaii | 3.00 (2.78 - 3.23) |
| Idaho | 2.00 (1.81 - 2.18) |
| Illinois | 1.41 (1.35 - 1.46) |
| Indiana | 2.92 (2.81 - 3.02) |
| Iowa | 3.10 (2.95 - 3.25) |
| Kansas | 1.87 (1.75 - 2.00) |
| Kentucky | 1.94 (1.83 - 2.04) |
| Louisiana | 1.18 (1.10 - 1.27) |
| Maine | 2.77 (2.56 - 2.98) |
| Maryland | 2.72 (2.61 - 2.83) |
| Massachusetts | 1.12 (1.06 - 1.18) |
| Michigan | 1.74 (1.67 - 1.80) |
| Minnesota | 2.56 (2.45 - 2.67) |
| Mississippi | 1.13 (1.03 - 1.23) |
| Missouri | 1.98 (1.89 - 2.07) |
| Montana | 2.14 (1.92 - 2.36) |
| Nebraska | 3.1 (2.9 - 3.3) |
| Nevada | 1.15 (1.04 - 1.27) |
| New Hampshire | 2.65 (2.43 - 2.87) |
| New Jersey | 1.92 (1.84 - 1.99) |
| New Mexico | 1.36 (1.23 - 1.49) |
| New York | 1.29 (1.25 - 1.33) |
| North Carolina | 2.42 (2.34 - 2.51) |
| North Dakota | 3.83 (3.47 - 4.18) |
| Ohio | 4.2 (4.11 - 4.29) |
| Oklahoma | 2.46 (2.33 - 2.59) |
| Oregon | 3.18 (3.04 - 3.32) |
| Pennsylvania | 2.17 (2.11 - 2.24) |
| Rhode Island | 2.84 (2.59 - 3.08) |
| South Carolina | 2.23 (2.12 - 2.34) |
| South Dakota | 2.35 (2.1 - 2.6) |
| Tennessee | 2.49 (2.39 - 2.59) |
| Texas | 2.94 (2.87 - 3.0) |
| Utah | 1.03 (0.91 - 1.15) |
| Vermont | 5.06 (4.63 - 5.49) |
| Virginia | 1.68 (1.61 - 1.76) |
| Washington | 3.08 (2.97 - 3.19) |
| West Virginia | 3.26 (3.07 - 3.46) |
| Wisconsin | 1.88 (1.79 - 1.96) |
| Wyoming | 2.25 (1.92 - 2.58) |
